# Choose Where You Live Carefully: Built Environment Differences in Children’s Cardiorespiratory Fitness and Cardiometabolic Risk

**DOI:** 10.3390/sports9020031

**Published:** 2021-02-21

**Authors:** Alan M. Nevill, Cézane Priscila Reuter, Caroline Brand, Anelise Reis Gaya, Jorge Mota, Jane Dagmar Pollo Renner, Michael J. Duncan

**Affiliations:** 1 Faculty of Education, Health and Wellbeing, University of Wolverhampton, Walsall WS1 3EZ, UK; a.m.nevill@wlv.ac.uk; 2 Graduate Program in Health Promotion, University of Santa Cruz do Sul, 96816-501 Santa Cruz do Sul/RS, Brazil; cezanereuter@unisc.br (C.P.R.); carolbrand@hotmail.com.br (C.B.); janerenner@unisc.br (J.D.P.R.); 3 School of Physical Education, Physiotherapy and Dance, Federal University of Rio Grande do Sul, 90690-200 Porto Alegre/RS, Brazil; anegaya@gmail.com; 4 Faculty of Sport, University of Porto, 4099-002 Porto, Portugal; jmota@fade.up.pt; 5 Sport, Exercise and Life Sciences Research Centre, Coventry University, Coventry CV1 5FB, UK

**Keywords:** urban, rural, fitness, health, paediatrics

## Abstract

Information regarding urban-rural differences in health indicators are scarce in Brazil. This study sought to identify rural-urban differences in cardiorespiratory fitness (CRF) and cardiometabolic risk (CMR) in Brazilian children and adolescents whilst controlling for the important confounding variables including social economic status (SES). This is a cross-sectional study developed with children and adolescents (n = 2250, age 11.54 ± 2.76) selected from a city in the south of Brazil. CRF was estimated using a 6-minute run/walk test. CMR scores were calculated by summing different cardiometabolic risk indicators. CRF was analysed assuming a multiplicative model with allometric body-size components. CMR differences in residential locations was assessed using Analysis of caovariance (ANCOVA) adopting SES, Body Mass Index (BMI), waist circumference (WC), age and fitness as covariates. Results indicated a main effect of location (*p* < 0.001) with children living a rural environment having the highest CRF, and children living in the periphery of towns having the lowest. Analysis also revealed significant main effects of location (*p* < 0.001) with children living a rural environment having the lowest CMR and children living in the centre of towns having the highest. Therefore, Brazilian children living in a rural environment appear to have superior health benefits.

## 1. Introduction

Cardiorespiratory fitness (CRF) remains an important marker of health status in children and adolescents [1]. Low levels of CRF in adolescence increases all-cause mortality risk in adulthood [2] and is associated with unfavourable cardiometabolic risk (CMR) in both children and adolescents [3,4,5]. Consequently, considerable efforts have been made to understand the correlates of CRF in children and adolescents as first steps to effectively target interventions for health benefit. Following the guidelines for physical activity practice (more than 60 min a day of moderate to vigorous physical activity) may increase CRF levels, along with different benefits for physical and mental health, including a reduction in CMR, and symptoms of anxiety and depression [6]. However, it is clear that not all individuals are aware of these guidelines, and even then, many do not adhere to them [7] In addition, obesity plays a critical role in the development of metabolic disorders in childhood, which is a worrying scenario considering the high prevalence of overweight and obesity in paediatric population, and the inaccurate perception of parents concerning their children’s weight status [8].

The built environment has emerged, over the last two decades, as an important consideration when examining health-related variables in children, adolescents and adults [9,10]. A considerable number of studies have examined differences in variables such as physical activity, weight status and physical fitness between individuals living in rural versus urban environments. The results of such studies are largely equivocal, in part due to the complex interactions between environmental and social factors that influence fitness and the fact that such interactions are specific to the particular geographical and environmental constraints where different populations reside [9]. There are also country and context specific variables which prior research has failed to fully consider. For example, in Brazil perceptions of violence relate to use of parks for leisure [11]. There have long been suggestions that children’s health-related fitness need to be studied across different geographic boundaries and in different climatic, economic and cultural contexts [12]. Several studies have examined differences in health and fitness parameters in children living in rural and urban areas [10,13,14,15]. However, data pertaining to this issue remain equivocal. McMurray, et al [14] reported that rural children in the USA had higher levels of adiposity but not CMR or CRF than their urban peers. Conversely Mamalakis et al. [13] reported higher adiposity in children from urban areas of Crete, compared to their rural peers. Sylejemani et al. [15] most recently reported significantly higher cardiorespiratory and muscular fitness in Macedonian children from rural compared to urban areas. One other study of note employed an allometric approach to examine rural-urban differences in different fitness parameters between rural and urban children in Greece. Tsimeas et al. [16] reported no differences in CRF between rural and urban children, although, some aspects of motor fitness did differ between rural and urban girls.

The equivocal nature of prior studies examining rural-urban differences in children’s physical fitness and CMR may be due to a number of reasons. Specific, climatic and environmental conditions between countries and regions mean that inferring findings from one region of the world to another is likely to lead to erroneous conclusions. Likewise, the equivocality of studies may be due, in part, to confounding by other variables. In particular, there seems to be a higher prevalence of low socioeconomic status (SES) individuals in rural areas [17]. Movement behaviour is also associated with low SES in children [18]. This would suggest that SES needs to be considered and accounted for when examining rural-urban differences in health and movement related variables such as fitness, physical activity and CMR in children and adolescents. To date, no study has examined rural-urban differences in Brazilian children and adolescents. Such information is key in putting in place geographically appropriate and country specific means to improve health related variables in young people. The current study sought to address key gaps in the literature by examining rural-urban differences in CRF and CMR in Brazilian children and adolescents whilst accounting for the important confounding variables including SES.

## 2. Materials and Methods

### 2.1. Study Design and Sample

This cross-sectional study was conducted with 2250 children and adolescents, aged 6 to 17 years (Mean age 11.54 ± 2.76), students from the city of Santa Cruz do Sul, Rio Grande do Sul—Brazil. Students were selected by conglomerate from 19 public and private schools (See Table 1). In 2004, a survey was conducted in this city, which indicated the number of schools (n = 50) and students (n = 17,688) enrolled. Then, a sample size calculation was made considering the population density of schoolchildren in all regions of the city (south, north, east, west, centre) including both private and public (municipal and state) schools. After that, schools were randomly selected and invited to form a cohort. From the present study, data were collected in 2016/2017 with a total sample size of 2250 participants. Only those schools who were originally involved in the 2004 survey were contacted to take part in this study, and thus could be considered a convenience sample. However, the sample is adequately powered as a posteriori power calculation indicated for ANCOVA analysis, to detect a small effect at 80% power and *p* = 0.05, a total sample size of 1634 would be required. The Human Research Ethics Committee of the University of Santa Cruz do Sul (UNISC) approved the study (certificate number 1.498.305). The schoolchildren’s parents or legal guardians signed free and informed consent forms.

### 2.2. Instruments and Procedures

Data were collected in the facilities of UNISC by trained researches. Weight and height were measured on the anthropometric scale with a coupled stadiometer (Filizola®, São Paulo, Brasil). Then, body mass index (BMI) was calculated by dividing body mass (in kilograms) by height (in square meters). Waist circumference (WC) was measured through an inelastic tape with a resolution of 1 mm (Cardiomed®) placed on the narrowest part of the trunk between the last rib and the iliac crest.

CRF was evaluated by the 6-minute run/walk test, following the procedures recommended by the Projeto Esporte Brasil (PROESP-BR) (Gaya, 2015) [19]. The participant should accomplish the greatest number of turns, running or walking, in a sports court with the perimeter marked with cones and the floor with indications of meters. The number of laps successfully completed, plus the additional distance achieved in children unable to complete a full lap at the end of the test, was calculated. Then, the estimation of CRF was obtained by multiplying the number of laps by meters covered, with subsequent calculation of the peak oxygen uptake (VO_2peak_) in mL/kg/min, following the Equation VO_2peak_ = 41.946 + 0.022 × (Test) − 0.875 × (BMI) + 2.107 × (Sex) [20]. The distance performed by the student in meters was used for the value of the “Test”, and the values of 1 and 0 for males and females, respectively. The estimated values from the 6MW test for VO_2peak_ in the current study were comparable to those previously reported for Brazilian children [20].

The area where participants lived and SES were reported by the parents/guardians of the participants using a questionnaire. For area of living they should indicate one of the following options (centre, periphery and rural). SES was classified according to Associação Brasileira de Empresas de Pesquisa (ABEP) [21], which considers the head of the household’s educational level and the number of certain items they have (car, washing machines, bathrooms, among others). The instrument called "Economic classification criteria Brazil”, from ABEP [21] is widely used in Brazil for the assessment of SES. The score derived from the questionnaire involves the number of items in the household (bathroom, automobile, motorcycle, electronic devices, and household appliances), whether the individual has a domestic employee, running water, a paved street, and also the level of education of the head of the family. The estimates for the class distributions are based on national probabilistic studies. The instrument does not, however, consider family income, as this parameter is not an efficient estimator of SES in Brazil [21]. Each answer was scored and the sum of these scores was used as a measure of the family’s social class. Participants were subsequently classified into eight distinct economic classes (A1, A2, B1, B2, C1, C2, D, and E). The choice of questionnaire instrument to assess SES was based on that recommended for use in the Brazilian context [21].

The systolic blood pressure (SBP) and diastolic blood pressure (DBP) were measured following the Brazilian guidelines for blood pressure measurement in children and adolescents using a sphygmomanometer (B-D®, aneroid, Heidelberg, Germany) with cuff suitable for the child’s arm circumference and stethoscope (Premium, Rappaport, Wuxi, Jiangsu, China). The evaluation took place with the participant seated and at rest.

Blood samples were collected after a 12-h fasting period, and the serum samples were stored at −112 °F until analysis. Concentrations of triglycerides, total cholesterol (TC), high-density lipoprotein (HDL-C), and glucose were determine. The tests were carried out through automated equipment Miura 200 (I.S.E., Rome, Italy) using commercial kits (DiaSysDiagnostic Systems, Holzheim, Germany).

Before analysis, CRF [VO_2max_], WC, TC/HDL-C ratio, and triglycerides were transformed by the natural logarithm. The variables WC, SBP, glucose, triglycerides, and TC/HDL-C ratio were standardized according to sex and age-specific international reference values using the Equation suggested by Stavnsbo et al. [22], as follows: Z-score= (X_Brazilian_ − X_International reference_)/SD_International reference_). Following this, a clustered CMR score was calculated by summing these variables and dividing by five.

### 2.3. Statistical Methods

A multiplicative model with allometric body-size components, originally proposed by Nevill and Holder [23] and recently adopted by Nevill et al. [24], was used to explore differences between categorical (e.g., sex, residential location) and associations with body-size variables, given by

VO_2max_ (mL·kg^−1^·min^−1^) = M^k1^ × H^k2^ × WC^k3^ exp(a + b·age + c·age^2^ + d·SES) × ε, (1)

where “a” is the constant intercept term, M = mass, H = height, WC=waist circumference, SES = socioeconomic status points and “ε” is a multiplicative, error ratio that assumes the error will be in proportion to VO_2max_ (mL·kg^−1^·min^−1^). The inclusion of height, mass and WC within the model is important as previous research has demonstrated that inclusion of all three of these anthropometric variables identifies people at risk of excess fatness and cardiometabolic risk more effectively than height and mass alone. Likewise, by using the logs of height, mass and WC as covariates, that ratio of these anthropometric variables can be ascertained (by taking the antilogs), and thereby indices such as Body Mass Index are included by proxy.

The model (Equation (1)) can be linearized with a log transformation (using Ln = log_e_). A linear regression analysis on Ln(VO_2max_) can then be used to estimate the unknown parameters in the log transformed model i.e., the transformed model (Equation (2)) is now additive that conforms with the assumptions associated with ordinary least squares and ANCOVA:

Ln(VO_2max_)= k_1_·Ln(M)+k_2_·Ln(H) + k_3_·Ln(WC) + a + b·age + c·age^2^ + d·SES + Ln(ε), (2)

where the residual errors Ln(ε) are assumed to be normally distributed and homoscedastic and the intercept “a” is allowed to vary by sex, age classification (child; 6 to 9 years vs. adolescents; 10 to 17 years) and location. Please note, in Equation (2), all the terms LnM, LnH, LnWC, age and age^2^ are covariates in the ANCOVA analysis. The intercept term “a” is used as a fixed factor. The Statistical Package for Social Sciences (SPSS v25, IBM Corp, Armonk, NY, USA) was used for all analysis.

## 3. Results

**Figure 1:**
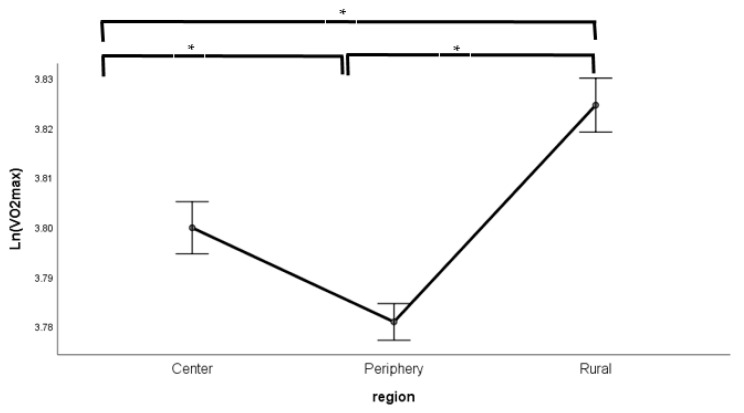
Differences in log-transformed VO2_max_ by location having controlled for body-size, sex, age and socioeconomic status (SES). (Data are mean ± SE), * *p* < 0.01.

**Figure 2:**
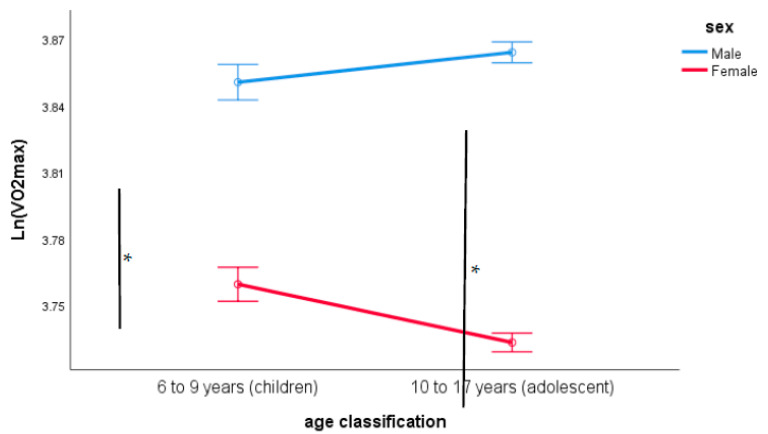
Differences in log-transformed VO_2max_ by sex and age group classification having controlled for body-size, sex, age and SES. (Data are mean ± SE), * *p* < 0.01.

The ANCOVA of log-transformed VO_2max_ identified the main effects of sex and location as significant (location and sex; both *p* < 0.001) plus the age classification-by-sex interaction (*p* < 0.001). The “location” main effect and the age classification-by-sex interaction can be seen in Figure 1 and Figure 2.

When fitting the log-transformed allometric model Equation (2) to VO_2max_, all three body-size covariates together with age (but not age^2^) and SES points were significant, see Table 2.

Note that the fitted mass (M) and height (H) exponents have opposite signs. The resulting product H^1.48^ × M^−0.58^ can be expressed as a ratio (H^1.48^/M^0.58^) or approximately (H^2.5^/M)^0.58^ not dissimilar to the inverse BMI (iBMI = H^2^/M). The WC exponent also confirms the detrimental effect of excess central adiposity associated with a child’s VO_2max_ (mL·kg^−1^·min^−1^).

**Figure 3:**
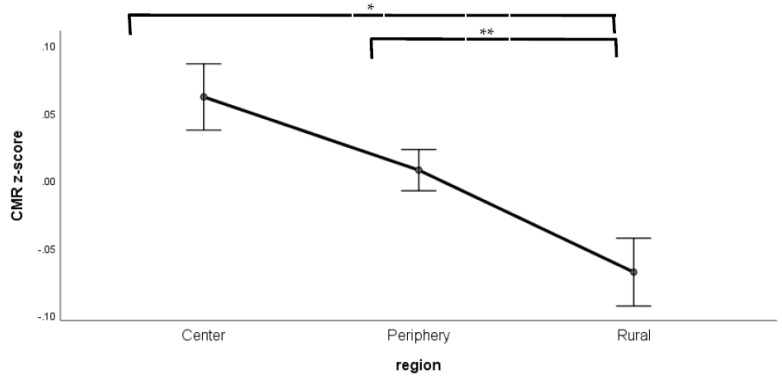
Differences in ardiometabolic risk (CMR) by location (*p* < 0.001) having controlled for the covariates of age, age^2^, sex, BMI, WC, VO_2max_ (mL·kg^−1^·min^−1^) and SES points. (Data are mean ± SE), * *p* < 0.01, ** *p* < 0.05.

The ANCOVA analysis of the CMR revealed significant main effects of location (*p* < 0.001), see Figure 3, but no difference due to sex or age classification having controlled for the covariates of age, age^2^, height, mass, WC, VO_2max_ (mL·kg^−1^·min^−1^) and SES points. The fitted covariate parameters are given in Table 3.

## 4. Discussion

The present study presents novel data illuminating the difference of living in rural or urban areas on CRF and CMR in Brazilian children and adolescents. Importantly, the present study also accounted for SES, an often-overlooked confounder in studies examining differences in health parameters between individuals living in rural or urban locations. The results of the present study demonstrate that, in this sample of Brazilian children and adolescents, place of residence has a significant and meaningful impact on both CRF and CMR. Accounting for SES, children and adolescents living in rural areas demonstrated higher values for CRF and lower CMR scores than children living in urban areas. The results of the current study add to the literature base on this topic, supporting some of the prior research on this topic whilst refuting others [10,13,14,15,16]. This is perhaps not surprising given that health behaviours influencing fitness and CMR differ from region to region and across countries [25,26]. The current study fills a gap in the literature by evaluating such rural–urban differences in Brazil, given that the effect of urbanization on cardiovascular disease and associated factors is country specific and studies are needed to identify the effects of urbanization on health for individual countries, before effective interventions strategies can be put in place [26]. It is also important to note that children in low to middle income countries face a higher burden of cardiovascular disease risk than their peers in high income countries and that urbanization has a less positive effect in such countries [26]. The results of the present study align with this assertion.

Drawing valid inference about differences (including residential) in CRF and CMR, can only be made if obvious confounding variables are incorporated into such analyses. For CRF, the effects of age, body size as well as SES are all essential confounders (see Equations (1) and (2), results given in Table 2). When predicting CRF, the body-size exponents combine to suggest a height-to-weight ratio somewhere between the inverse BMI (H^2^/M) and the reciprocal ponderal index (H^3^/M) is the optimal body shape associated with CRF. Indeed by incorporating the WC term into the model (Equation (1)), we reveal that WC provides a valuable new insight. i.e., a new “third” dimension associated with predicting CRF as well as excess fat [24]. Note that the SES parameter in Table 2 is negative indicating that the children from more affluent parents are less fit.

In interpreting the results of the current study it is also important to contextualise the findings related to the geographical area from which participants were drawn. Santa Cruz do Sul is a midsized city located in the south of Brazil, with a population of 129,427 inhabitants, and an area of 733.4 km². The majority of residents live in urban areas or on the periphery, with approximately 12,000 living in rural areas. The rural population are mainly engaged in agriculture, including the cultivation of tobacco and soy beans, and with a culture of families living in such areas all being actively involved in the industry, which may impact habitual physical activity levels. There are a number of suggestions as to why children and adolescents living in urban areas would present poorer CRF and CMR including more urban environments constraining opportunities for physical activity and promoting less positive dietary habits, including greater incidence of diets high in salt, sugar and fat [25,26]. Such rural–urban differences, as a consequence of unhealthy diets and urban built environments that constrain physical activity have been particularly identified for low to middle income countries [27]. This is principally because in low to middle income countries there is more unplanned urbanization in cities; such urban living can remove the autonomy of individuals to make healthy choices, via dominant influences encouraging the adoption of unhealthy behaviours [28]. Specifically in the context of Brazil, an inverse association between crime safety and walking for leisure has been found in prior research, as well as perceptions of road safety and distance to green space explaining a significant (16%) amount of the variance in park use for leisure [8]. An important observation from the results of the current study occurred when considering covariates. The data demonstrate a benefit of being physically fit (higher cardiorespiratory endurance) on CMR for children with a higher BMI. Irrespective of rural-urban residence higher CRF is a positive influence for children and adolescents irrespective of weight status. The current results therefore also support the fit-but-fat phenomenon [29,30]. Although there is some controversy regarding this paradox, as recently suggested by Valenzuaela et al. [31], which indicated that although physical activity may reduce the detrimental effects of adiposity on cardiovascular risk, excess body weight is strongly associated with an increase in the prevalence or risk factors, such as hypertension and hypercholesterolaemia. Indeed, our findings indicated that when predicting CMR in Table 3, the positive slope parameter of BMI (B = 0.013) is similar to the negative slope parameter of VO_2max_ (B = −0.012), suggesting that if a child were to gain one unit of BMI (kg·m^−2^), the increase in CMR will be compensated by the same child also increasing their VO_2max_ by one unit (mL·kg^−1^·min^−1^).

The present study does have limitations that should be acknowledged. The cross-sectional nature of the data prevents establishing causality. In addition, although we observe rural-urban differences in health outcomes (CRF and CMR), we do not have data relating to the behaviours that may lead to the aforementioned health outcomes. As lifestyle factors have been identified as the mechanism by which rural-urban differences in cardiovascular disease risk and its correlates develop [26] having data on dietary and physical activity habits would have been useful in further identifying why such rural-urban differences exist. Likewise, it would be useful to understand context specific variables such as parental perception of violence and crime. In addition, the role of environmental factors that may relate to opportunities to engage in physical activity, such as distance from home to the nearest park or square, would also be useful in shaping understanding of rural-urban differences in health outcomes. Alongside this, considering that parents’ physical activity may influence their children´s physical activity, this should also be investigated in the context of the role of place of residence on CMR and CRF [32]. These aspects should be considered as a future research focus. Finally, it is difficult to extend the current findings to the whole of Brazil. Considering that Brazil is a country with very large dimensions, and with cultural and geographical differences across regions, we should be cautious when extrapolating present results to other regions of the country. Rather, the results of the present study should be considered in the context of midsized cities where there are defined urban, periphery and rural geographical distinctions.

## 5. Conclusions

In conclusion, the present study suggests that place of residence has significant impacts on health in children and adolescents from a city in the southern of Brazil. Specifically, living in a rural environment benefits CRF and CMR, whereas living in an urban environment is associated with poorer CRF and higher CMR even after accounting for SES. There is thus a need for more effective public health practice and policy related to health effects of the built environment for children and adolescents in Rio Grande do Sul—Brazil.

## Figures and Tables

**Table 1:** The number of children by age, sex and location.

Age	Male			Female			Total
	Centre	Periphery	Rural	Centre	Periphery	Rural	
6	6	6	0	8	15	0	35
7	25	31	12	17	41	19	145
8	27	41	12	35	52	20	187
9	23	69	26	31	71	17	237
10	23	62	26	34	77	31	253
11	23	61	27	31	89	27	258
12	22	74	33	32	82	25	268
13	14	69	30	24	89	30	256
14	14	50	30	16	95	26	231
15	19	36	15	36	64	17	187
16	24	31	10	22	26	15	128
17	12	11	6	18	11	7	65
-	232	541	227	304	712	234	2250

**Table 2:** The fitted parameters (±SE), t-scores and 95% confidence intervals (CI) associated with predicting log-transformed VO_2max_ using Equation (2).

Parameter	B	Std. Error	t	Sig.	95% Confidence Interval	
					Lower Bound	Upper Bound
Intercept	5.948	0.089	66.953	<0.001	5.774	6.122
age	0.007	0.002	3.944	<0.001	0.003	0.010
LnMass	−0.580	0.025	−23.399	<0.001	−0.629	−0.531
LnH	1.480	0.059	25.131	<0.001	1.364	1.595
LnWC	−0.149	0.035	−4.237	<0.001	−0.218	−0.080
SES points	−0.002	0.000	−3.921	<0.001	−0.003	−0.001
Male	0.130	0.010	12.574	<0.001	0.110	0.151

R Squared = 0.619 (Adjusted R Squared = 0.617). Ln. log_e_; HT. Height; WC. Waist circumference; SES. Socioeconomic status.

**Table 3:** The fitted parameters (±SE), t-scores and 95% confidence intervals (CI) associated with predicting cardiometabolic risk (CMR) having controlled for the covariates of age, age^2^, sex, BMI, WC, VO_2max_ (mL·kg^−1^·min^−1^) and SES points.

Parameter	B	Std. Error	t	Sig.	95% Confidence Interval	
					Lower Bound	Upper Bound
Intercept	−0.500	0.375	−1.333	0.183	−1.237	0.236
Age	−0.228	0.047	−4.852	<0.001	−0.320	−0.136
age^2^	0.008	0.002	4.087	<0.001	0.004	0.011
BMI	0.013	0.007	2.020	0.043	0.000	0.026
WC	0.033	0.002	14.743	<0.001	0.029	0.037
VO_2_max	−0.012	0.003	−3.572	<0.001	−0.019	−0.005
SES points	0.005	0.002	2.104	0.036	0.000	0.009
Male	−0.062	0.029	−2.114	0.035	−0.120	−0.005

R Squared = 0.401 (Adjusted R Squared = 0.398). BMI. Body mass index; WC. Waist circumference; VO_2max_. maximum oxygen consumption; SES. Socioeconomic status.

## Data Availability

The data presented in this study are available on request from the corresponding author.
